# Effect of Sodium Tanshinone IIA Sulfonate Injection on Blood Lipid in Patients With Coronary Heart Disease: A Systematic Review and Meta-Analysis of Randomized Clinical Trials

**DOI:** 10.3389/fcvm.2021.770746

**Published:** 2021-11-24

**Authors:** Hufang Zhou, Ying Zhao, Wenhua Peng, Wenbo Han, Zichen Wang, Xiaoxia Ren, Dayang Wang, Guozhong Pan, Qian Lin, Xian Wang

**Affiliations:** ^1^Cardiovascular Diseases Center, Dongzhimen Hospital, Beijing University of Chinese Medicine, Beijing, China; ^2^Dongfang Hospital, Beijing University of Chinese Medicine, Beijing, China; ^3^Beijing University of Chinese Medicine Third Affiliated Hospital, Beijing, China; ^4^Changping District Hospital of Integrated Traditional Chinese and Western Medicine, Beijing, China

**Keywords:** Sodium Tanshinone IIA Sulfonate, blood lipid, coronary heart disease, randomized controlled trials, systematic review, meta-analysis

## Abstract

**Background:** Lipid-lowering therapy is very important in secondary prevention of coronary heart disease (CHD). In many clinical trials, it has been found that Sodium Tanshinone IIA Sulfonate Injection (STS) have a lipid-lowering effect while reducing major cardiovascular events in patients with CHD. However, up to now, there is no system review on the effectiveness and safety of STS affecting blood lipids.

**Purpose:** The aim of this review is to systematically assess the effects of STS on blood lipid levels in patients with CHD.

**Methods:** Until Mar 2021, five databases (PubMed, EMBASE, Cochrane Library, China National Knowledge Infrastructure, and Wanfang Database) were searched for randomized controlled trials (RCTs) about STS treating patients with CHD. Risk bias was assessed for included studies according to Cochrane handbook. The primary outcome was total cholesterol (TC). The secondary outcomes were triglycerides (TG), low-density lipoprotein cholesterol (LDL-c), high-density lipoprotein cholesterol (HDL-c), and adverse events (AEs).

**Results:** A total of 27 trials including 2,445 CHD patients met the eligibility criteria. Most trials had high risks in random sequence generation, allocation concealment, blinding of patients and personal, blinding of outcome assessment. Meta-analysis showed that STS significantly reduced plasma TC levels [MD = −1.34 mmol/l 95% CI (−1.59, −1.09), *p* < 0.00001, *I*^2^ = 98%], TG levels [MD = −0.49 mmol/l 95% CI (−0.62, −0.35), *p* < 0.00001, *I*^2^ = 97%], LDL-c levels [MD = −0.68 mmol/l (−0.80, −0.57), *p* < 0.00001, *I*^2^ = 96%], increased HDL-c levels [MD = 0.26 mmol/l (0.15, 0.37), *p* < 0.00001, *I*^2^ = 97%], without increasing the incidence of AEs [RR = 1.27 95% CI (0.72, 2.27), *p* = 0.94, *I*^2^ = 0%] in patients with CHD.

**Conclusion:** STS can safely and effectively reduce plasma TC, TG and LDL-c levels in patients with CHD, and improve plasma HDL-c levels. However, these findings require careful recommendation due to the low overall quality of RCTs at present. More multi-center, randomized, double-blind, placebo-controlled trials which are designed follow the CONSORT 2010 guideline are needed.

**Graphical Abstract:**
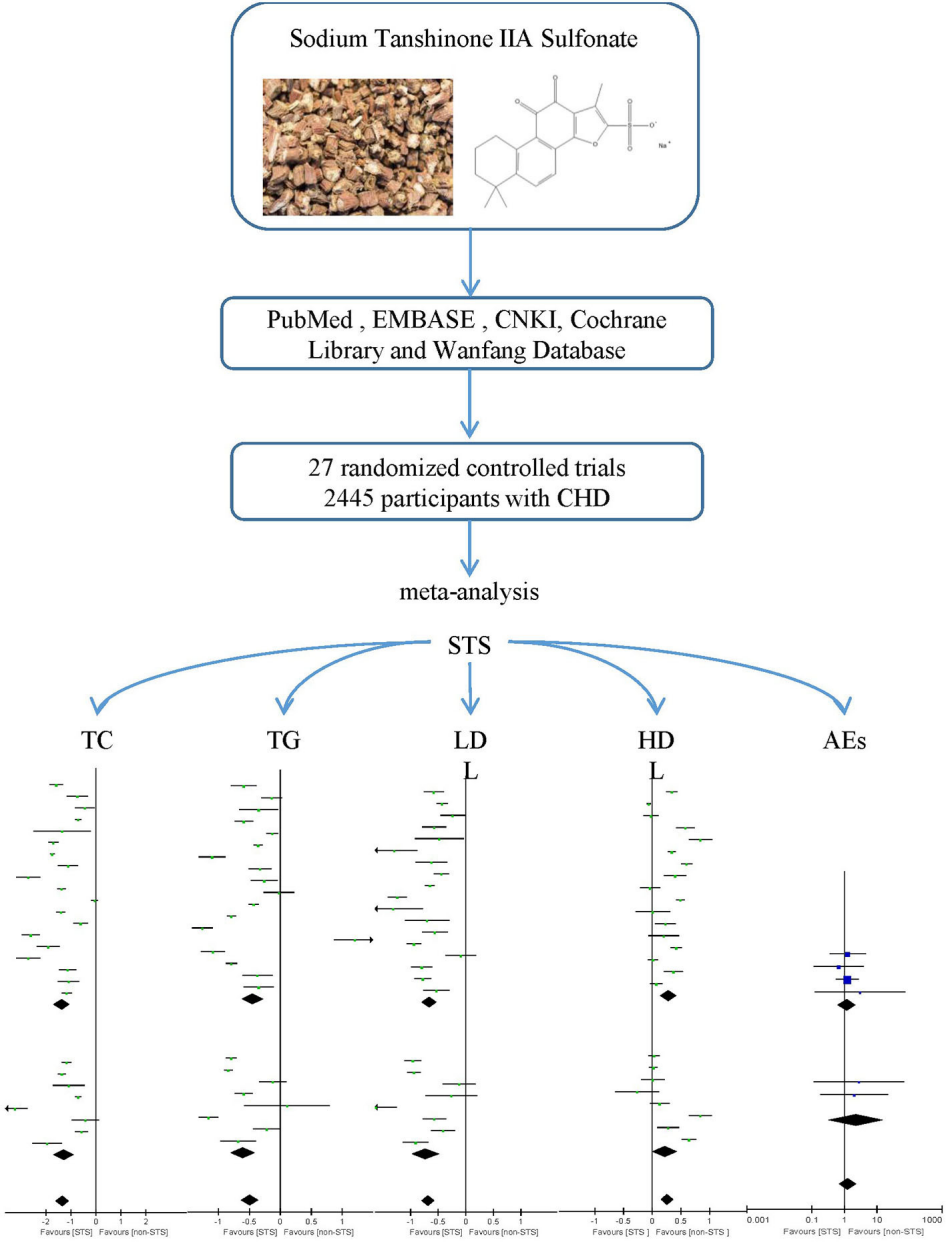
A Systematic Review and Meta-analysis was performed to investigate the effect of Sodium Tanshinone IIA Sulfonate Injection on blood lipid in patients with coronary heart disease.

## Introduction

Lipid-lowering therapy, such as Statins, is very important in secondary prevention of CHD. Statins play an irreplaceable role as first-line lipid-lowering drugs for secondary prevention of CHD. Clinical practice guidelines recommend that all non-ST-elevation acute coronary syndromes (NSTE-ACS) patients should be treated with high-intensity statins as soon as possible after admission (1). Statins use should also be considered in patients with confirmed stable coronary heart disease (SCAD) or chronic coronary syndrome (CCS) regardless of LDL-c levels (2). However, the use of statin lipid-lowering drugs is susceptible to clinical side effects, including statin-related muscle symptoms (SAMS), as well as the risks of liver disease, diabetes, cognitive impairment and hemorrhagic stroke (3, 4). Guidelines also recommend that dietary supplements, including phytosterols, should be considered for high-risk CHD patients with statin intolerance.

Traditional Chinese medicine, which has a history of thousands of years, provides us with a large number of complementary and alternative drugs. In recent years, red yeast rice extract (5), berberine (6), ryegrass extract (7), policosanol (8), astaxanthin (9) and other drugs were found to have significant lipid-lowering effects in clinical and basic research, and some of them had been used to assist in clinical lipid-lowering therapy. Salvia miltiorrhiza Bunge is one of the most commonly used herbs to accelerate blood circulation in China for treating cardiovascular diseases (CVDs) and other circulatory disturbance-related diseases (10). Tanshinone IIA is one of the most pharmacologically active ingredients extracted from Salvia miltiorrhiza Bunge and is considered as a promising natural cardioprotective agent which can reduce the levels of many inflammatory factors related to atherosclerosis progression, such as C-reactive protein (CRP), interleukin-6 (IL-6), tumor necrosis factor-alpha (TNF-α), vascular cell adhesion molecule-1 (VCAM-1), CD40, monocyte chemotactic protein-1 (MCP-1) and matrix metalloproteinase-9 (MMP-9) (10–13). Because of low oral bioavailability of tanshinone IIA Sulfonate, intravenous sodium tanshinone IIA sulfate (STS) has been developed. Tanshinone IIA preparation is one of the most widely used medical treatment for patients with CHD in China. In many clinical trials in China, STS has been found to have a lipid-lowering effect while reducing major cardiovascular events in patients with CHD (14). However, up to now, there has been no systematic review on the efficacy and safety of STS affecting blood lipids in patients with CHD. Therefore, the purpose of this systematic review and meta-analysis is to systematically assess the efficacy and safety of STS on blood lipid levels in patients with CHD.

## Methods

The review protocol was registered at PROSPERO (No: CRD42018109290, https://www.crd.york.ac.Uk/prospero/). This study was conducted according to Cochrane Handbook for Systematic Reviews of Interventions (15) and was reported according to Preferred Reporting Items for Systematic reviews and Meta-Analyses (PRISMA) (16).

### Literature Search

The literature searches were conducted in the following five databases: PubMed (1966 to 2021), EMBASE (1974 to 2021), China National Knowledge Infrastructure (CNKI) (1979 to 2021), Cochrane Library (2000 to 2021), and Wanfang Database (1985 to 2021). In order to check the whole, we searched all the literatures concerning STS. The main search terms was “Sodium Tanshinone IIA Sulfonate.” There are no restrictions on language, date of publication or status of publication. All searches ended on March 4, 2021.

### Eligibility Criteria

Original literatures were included if they met the following inclusion criteria: (1) Types of studies (S): RCTs with or without blinding; (2) Types of participants (P): Patient with CHD, including patients with CCS and acute coronary syndrome (ACS); (3) Types of interventions (I): STS with or without lipid-lowering drugs (LLDs); (4) Types of comparators (C): placebo, no drug or LLDs; intervention and control groups that used routine therapy (RT) simultaneously; (5) Types of outcome measures (O): any of the lipid profile parameters (including TC, LDL-c, HDL-c, TG and AEs). Exclusion criteria: (1) Duplicated publications; (2) Trials whose methods of allocation use date of birth, date of admission, hospital numbers, or alternation; (3) Use of other herbal medicine or extracts either in intervention or control group; (4) Lack of baseline or related lipid information.

### Data Extraction

Data extraction was performed independently by two reviewers (Zhou HF, Lin Q). The extracted data included: authors, title of study, year of publication, sample size, PICOS details and treatment duration. We defined TC as the primary outcome and LDL-c, HDL-c, TG and AEs as secondary outcomes. AEs were defined as a composite of events including headache, dizziness, gastrointestinal reaction, liver function damage, allergy, renal function damage and bleeding.

### Risk of Bias Assessment

Two reviewers (Zhou HF, Lin Q) independently assessed the risk of bias for each included trial according to the Cochrane Handbook for Systematic Reviewers of Interventions version 5.1.0 (15). The items included random sequence generation (selection bias), allocation concealment (selection bias), blinding of participants and personnel (performance bias), blinding of outcome assessment (detection bias), incomplete outcome data (attrition bias), selective reporting (reporting bias), and baseline data comparability (other bias). Each item was categorized as low/unclear/high risk of bias. Disagreements were resolved by discussion, with involvement of a third review author (Wang X) when necessary. In addition, we used the grading of recommendations assessment, development, and evaluation (GRADE) approach to evaluate the quality of included evidences.

### Data Analysis

If the index to be analyzed were continuous variables, we selected mean difference (MD) as the effect scale. If it were binary variables, we selected risk ratio (RR). All results were presented with 95% confidence intervals (CI). Heterogeneity among trials was assessed by Cochrane's Q test and I-squared statistic. According to Cochrane Handbook for Systematic Reviews of Interventions, the scale of *I*^2^ had a range of 0–100% and values on the order of 0–40%, 30–60%, 50–90%, and 75–100% were considered might not be important, may represent moderate heterogeneity, may represent substantial heterogeneity and considerable heterogeneity, respectively (15). For heterogeneous studies, we adopted use a random-effects model to estimate the overall effect instead of a fixed-effect model, because random-effects models assess the outcomes of the study according to within-trial as well as between-trial variance (17), thus providing more conservative results. Furthermore, meta-regression analysis was used to explore the sources of heterogeneity. The sensitivity analysis was also performed by removing each study one at a time to evaluate the stability of the results. The Trial Sequential Analysis (TSA) was used to determine the robustness of on primary outcome and calculate the required information size (RIS) in the meta-analysis (18). Subgroup analysis was performed according to various types of interventions (with or without LLDs). The publication bias was detected by the funnel plot, the Begger's test and the Egger's test (19).

## Results

### Study Selection

**Figure 1:**
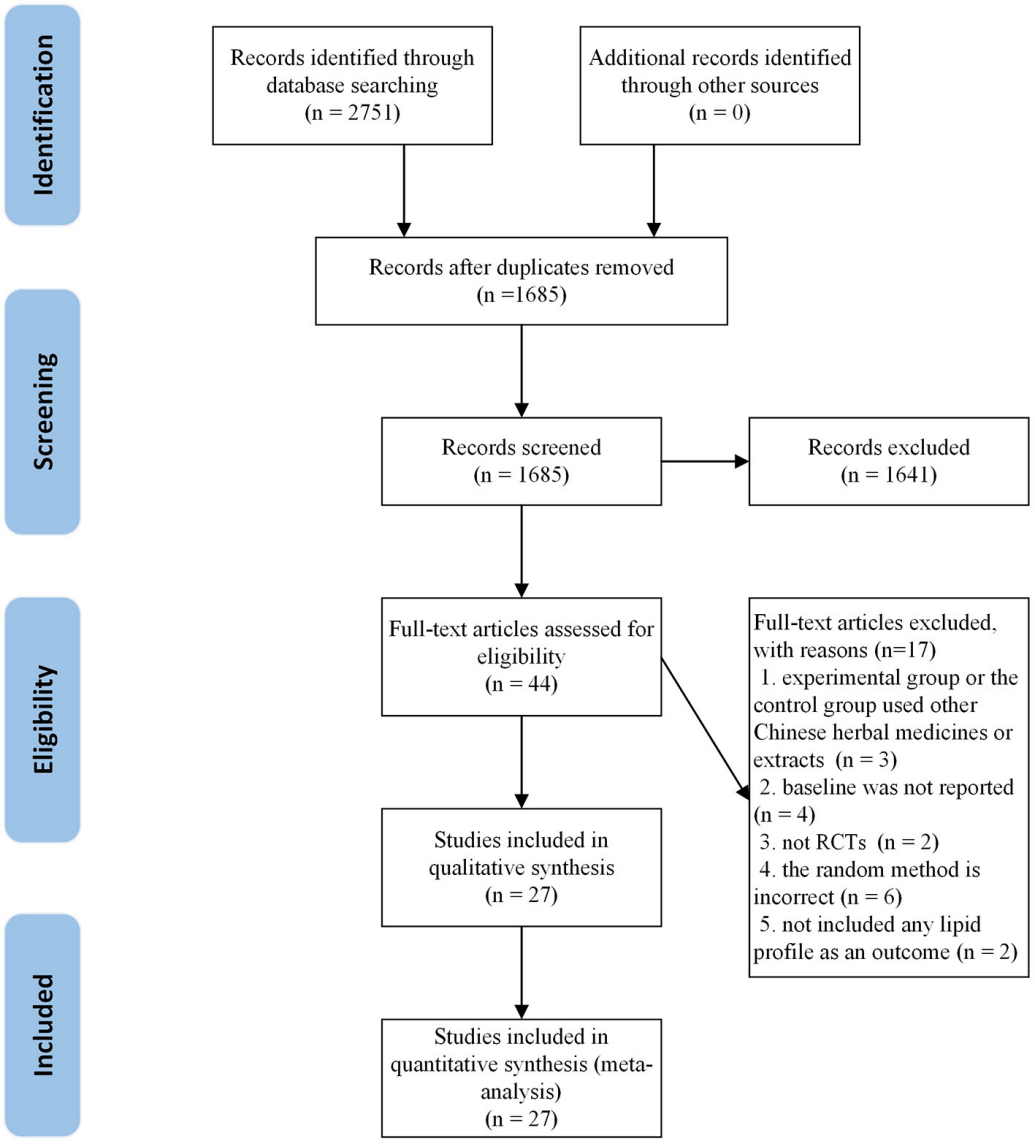
The flow chart of the study selection process showing how to screen eligible randomized controlled trials.

A total of 2,751 articles were retrieved from the initial search. After deleting the duplicate literature, 1,685 articles remained. After reading the title and abstract, 1,641 articles were excluded and 44 articles were screened in detail. By reading the full text of the remaining 44 articles, 17 articles were excluded, which did not meet our inclusion criteria: two were not RCTs (20, 21); three were combined with other herbs or extracts (22–24); two did not include any blood lipids profile in the outcome (25, 26); four did not report baseline (27–30) and six used nonstandard randomization methods (31–36). Finally, 27 trials (37–63) were qualified and included in the meta-analysis. The flow chart of literature screening is as follows (Figure 1).

### Study Characteristics

The 27 RCTs were all from China and single-center trials. The 2,445 patients recruited (1,228 in the trial group and 1,217 in the control group) were all hospitalized patients. The average age ranged from 47 to 72 years. Most studies recruited more male patients. Three studies (37–39) included patients with ACS; Three studies (40–42) included patients with acute myocardial infarction (AMI); Two studies (43, 44) included patients with unstable angina pectoris (UAP) and one study included CHD patients with hyperuricemia (45). The remaining studies only showed that patients with CHD or myocardial ischemia are included.

Except for one study (46), which was based on oral aspirin, the remaining studies were based on secondary prevention of CHD. Eight studies (40, 41, 46–51) did not use statins in combination. Seven studies (42, 44, 52–56) clearly stated that atorvastatin was used in combination; Three studies (45, 57, 58) stated that simvastatin was used in combination; Nine studies (37–39, 43, 59–63) only stated the use of statins, but did not specify which statins were used; Other studies stated that no statins were used in combination. The dosage of STS ranged from 40 to 80 mg per day. The intervention duration ranged from 1 week to 1 month. Most studies reported in detail the results of blood lipids. All 27 included RCTs provided data for TC, 26 RCTs provided data for TG and LDL-c and 25 RCTs provided data for HDL-c. Of the 27 RCTs, 15 studies (37–42, 44, 48, 50–52, 54–56, 60) did not report information about AEs. Six trials (43, 46, 53, 57, 59, 61) reported no AEs and the remaining six trials (45, 47, 49, 58, 62, 63) reported a total of 39 AEs. Characteristics of the trials and the detail of PICOS are shown in Table 1.

**Table 1:** Characteristics of the included RCTs and the detail of PICOS.

**Study ID**	**Participants**	**No. of participants (I/C)**	**Age (years)**	**Male (%)**	**Interventions**	**Comparators**	**Duration**
Fang et al., (63)	CHD	33/33	I: 58.6 ± 4.8	I: 17(51.52%)	STS 80 mg qd; statin; RT	statin; RT	14D
			C: 57.9 ± 5.4	C: 16(48.48%)			
Yan et al., (58)	CHD	36/36	I: 57.84 ± 7.63	I: 23 (63.89%)	STS 60 mg qd; Simvastatin 10 mg qn; RT	Simvastatin 10 mg qn; RT	1M
			C: 57.76 ± 7.82	C: 21 (58.33%)			
Zhang (45)	CHD with hyperuricemia	50/50	I: 61.70 ± 6.89	I: 35 (70.00%)	STS 60 mg qd; Simvastatin 10 mg qn; RT	Simvastatin 10 mg qn; RT	14D
			C: 61.41 ± 6.85	C: 33 (66.00%)			
He et al., (54)	CHD	29/29	I: 56.6 ± 16.1	I: 21 (72.41%)	STS 60 mg qd; Atorvastatin; RT	Atorvastatin; RT	14D
			C: 53.9 ± 14.5	C: 17 (58.62%)			
Li et al., (55)	CHD	50/50	I: 66 ± 5	I: 29 (58.00%)	STS 80 mg qd; statin; RT	Atorvastatin 10mg qn; RT	1M
			C: 64 ± 5	C: 28(56.00%)			
Yang (60)	CHD	52/52	I: 67.64 ± 9.46	I: 33 (63.46%)	STS 50 mg qd; statin; RT	statin; RT	14D
			C: 68.42 ± 9.32	C: 32 (61.54%)			
Cao et al., (61)	CHD	49/49	I: 61.49 ± 9.14	I: 33 (67.35%)	STS 60 mg qd; statin; RT	statin; RT	28D
			C: 62.59 ± 8.21	C: 30 (61.22%)			
Zhu et al., (62)	CHD	48/48	I: 65.3 ± 8.8	I: 28 (58.33%)	STS 60 mg qd; statin; RT	statin; RT	28D
			C: 64.7 ± 8.2	C: 26 (54.17%)			
Lin et al., (39)	ACS	49/49	I: 55.98 ± 5.49	I: 30 (61.22%)	STS 50 mg qd; statin; RT	statin; RT	14D
			C: 56.44 ± 5.86	C: 32 (65.31%)			
Guo (56)	CHD	50/50	I: 60.53 ± 9.42	I: 33 (66.00%)	STS 60 mg qd; Atorvastatin 20 mg qn; RT	Atorvastatin 20 mg qn; RT	14D
			C: 60.14 ± 8.43	C: 31 (62.00%)			
Jin (44)	UAP	50/50	I: 66.24 ± 8.3	I: 35 (70.00%)	STS 60 mg qd; Atorvastatin 20 mg qn; RT	Atorvastatin 20 mg qn; RT	14D
			C: 64.35 ± 9.4	C: 38 (76.00%)			
He et al., (57)	CHD	49/49	I: 64.19 ± 5.82	I: 28 (57.14%)	STS 60 mg qd; Simvastatin 10 mg qn; RT	Simvastatin 10 mg qn; RT	1M
			C: 62.31 ± 6.37	C: 30 (61.22%)			
Gao et al., (52)	CHD	46/46	I: 58.6 ± 8.9	I: 29 (63.04%)	STS 60 mg qd; Atorvastatin 20 mg qn; RT	Atorvastatin 20 mg qn; RT	14D
			C: 57.2 ± 8.1	C: 27 (58.70%)			
Xun et al., (53)	CHD	80/80	I: 60.12 ± 9.47	I: 37 (46.25%)	STS 80 mg qd; Atorvastatin 10 mg qn; RT	Atorvastatin 10 mg qn; RT	30D
			C: 58.75 ± 9.25	C: 38 (47.50%)			
Li et al., (38)	ACS	30/30	unclear	unclear	STS 80 mg qd; statin; RT	statin; RT	7D
Wang and Zheng (42)	AMI	46/46	unclear	unclear	STS 60 mg qd; Atorvastatin; RT	Atorvastatin; RT	7D
Huang (59)	CHD	30/30	I: 65.77 ± 8.90	I: 14 (46.67%)	STS 40 mg qd; statin; RT	statin; RT	14D
			C: 65.53 ± 7.78	C: 10 (33.33%)			
Zhang et al., (37)	ACS	32/32	I: 60 ± 7	I: 24 (75.00%)	STS 60 mg qd; statin; RT	statin; RT	10D
			C: 58 ± 9	C: 22 (68.75%)			
Ge et al., (43)	UAP	30/30	I: 57 ± 7	I: 19 (63.33%)	STS 60mg qd; statin; RT	statin; RT	15D
			C: 58 ± 7	C: 20 (66.67%)			
Zhao et al., (46)	CHD	39/39	I: 47.45 ± 2.13	I: 21 (53.85%)	STS 80mg qd; Aspirin 50 bid	no drug; Aspirin 50 bid	14D
			I: 51.23 ± 2.72	I: 19 (48.72%)			
Fan and Yang (41)	AMI	47/42	I: 59.7 ± 5.6	I: 26 (55.32%)	STS 60mg qd; RT	no drug; RT	7D
			C: 58.9 ± 4.4	C: 23 (54.76%)			
Wei and Shen (50)	CHD	60/60	I: 49.18 ± 12.50	I: 38 (63.33%)	STS 60mg qd; RT	no drug; RT	14D
			I: 52.36 ± 10.18	I: 36 (60.00%)			
Zhang et al., (40)	AMI	48/48	unclear	unclear	STS 40-80mg qd; RT	no drug; RT	7D
Gao et al., (51)	CHD	30/30	unclear	unclear	STS 60mg qd; RT	no drug; RT	14D
Cheng and Zhang (49)	CHD	60/60	I: 67.5 ± 6.1	I: 36 (60.00%)	STS 60mg qd; RT	no drug; RT	14D
			C: 66.2 ± 4.5	C: 38 (63.33%)			
Guo and Sun (48)	CHD	65/62	unclear	I: 35 (53.85%)	STS 50mg qd; RT	no drug; RT	14D
				C: 34 (54.84%)			
Tao and Jia (47)	CHD	40/37	I: 72 ± 10	I: 27 (67.50%)	STS 80mg qd; RT	no drug; RT	14D
			C: 74 ± 8.5	C: 25 (67.57%)			

*I, Intervention; C, control; RT, routine therapy; D, days; M, months*.

### Risk of Bias in Individual Studies

**Figure 2:**
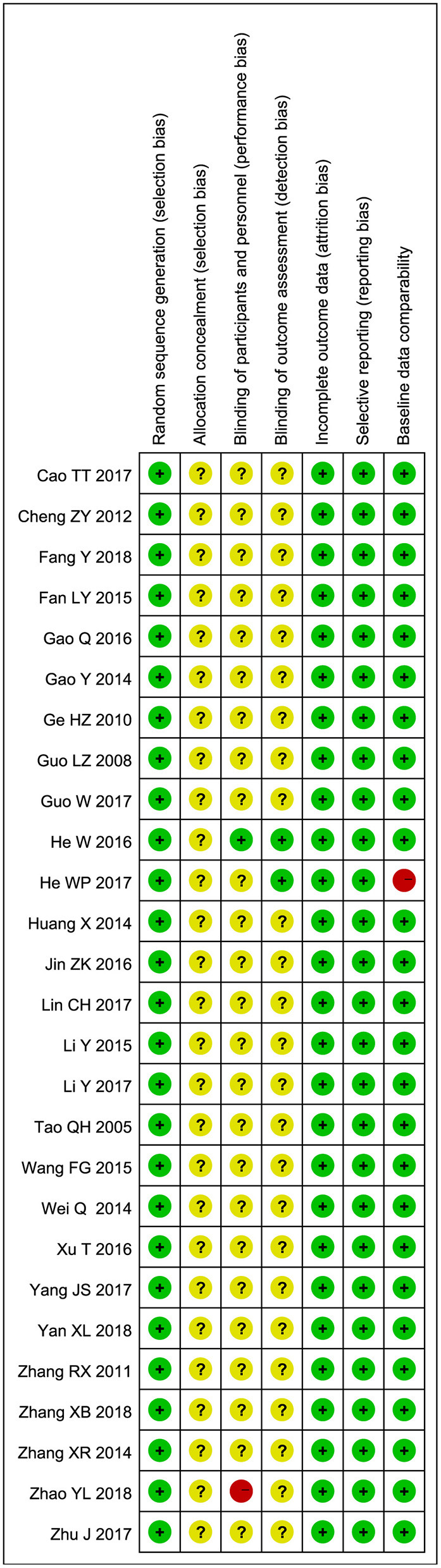
Risk of bias summary.

Randomization had been mentioned in all studies. Fifteen of them explicitly described methods of randomization, such as random number tables or dichroic spheres. None of the studies mentioned allocation concealment. Except for two studies, none of the other studies mentioned the blind method. One study made it clear that no blind method was used, and another made it clear that blind method was used. All studies have a low-risk selective reporting bias. All included studies were baseline comparable. The Risk of bias summary is shown in Figure 2. Supplement one (Supplementary Tables S1, S2) summarized the level of evidence for the studies included and indicated that the overall quality of the evidence was very low.

### Overall Results of Meta-Analysis

In addition to AEs, the remaining meta-analysis showed a high degree of heterogeneity among studies. Therefore, the meta-analysis of AEs adopted a fixed-effect model, while the remaining meta-analysis used a random-effect model.

**Figure 3:**
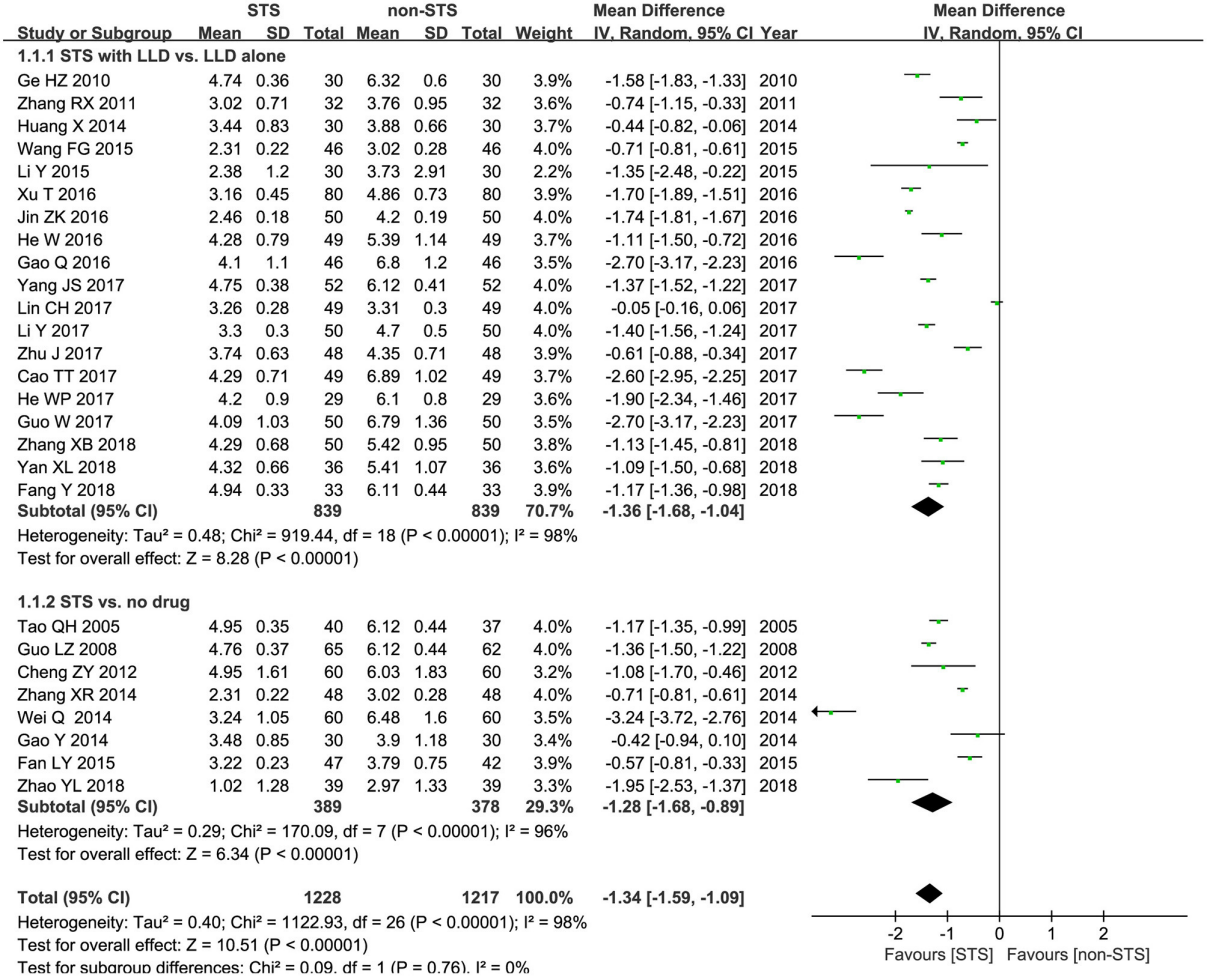
Forest plot of comparison: STS vs. non-STS in TC.

**TC**: As shown in Figure 3, meta-analysis of 27 studies showed that STS significantly reduced plasma TC levels in patients with CHD [MD = −1.34 mmol/l, 95% CI (−1.59, −1.09), *P* < 0.00001, *I*^2^ = 98%]. Subgroup analysis revealed that STS significantly lowered TC levels when used alone compared to blank control [MD = −1.28 mmol/l, 95% CI (−1.68, −0.89), *P* < 0.00001, *I*^2^ = 96%], or used in combination with LLDs compared to LLDs [MD = −1.36mmol/l, 95% CI (−1.68, −1.04), *P* < 0.00001, *I*^2^ = 98%].

**Figure 4:**
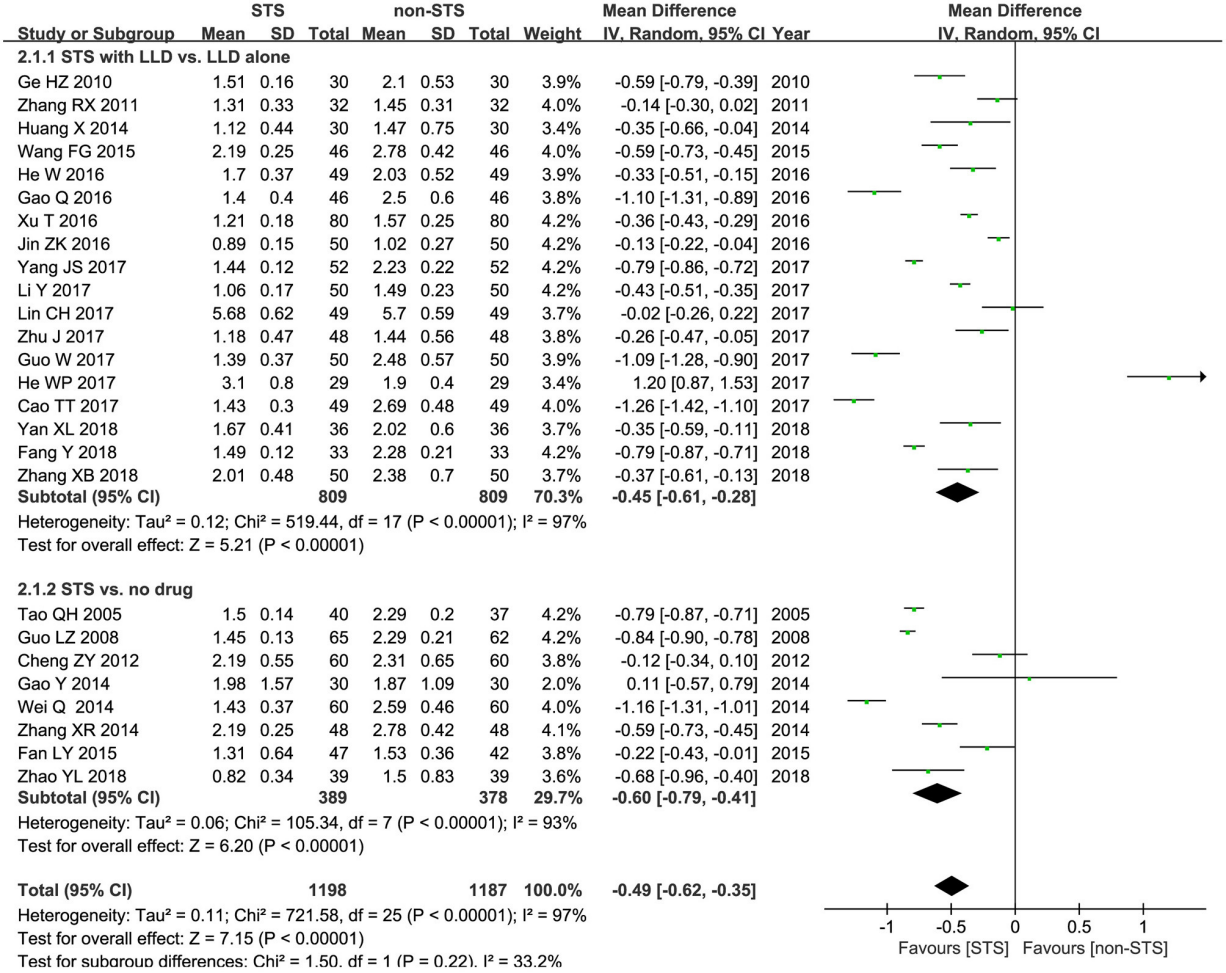
Forest plot of comparison: STS vs. non-STS in TG.

**TG**: As shown in Figure 4, pooled results of 26 studies revealed that STS significantly reduced plasma TG levels in patients with CHD [MD = −0.49 mmol/l, 95% CI (−0.62, −0.35), *P* < 0.00001, *I*^2^ = 97%). Subgroup analysis revealed that STS significantly lowered TG levels when used alone compared to blank control [MD = −0.60 mmol/l, 95% CI (−0.79, −0.41), *P* < 0.00001, *I*^2^ = 97%], or used in combination with LLDs compared to LLDs [MD = −0.45 mmol/l, 95% CI (−0.61, −0.28), *P* < 0.00001, *I*^2^ = 97%].

**Figure 5:**
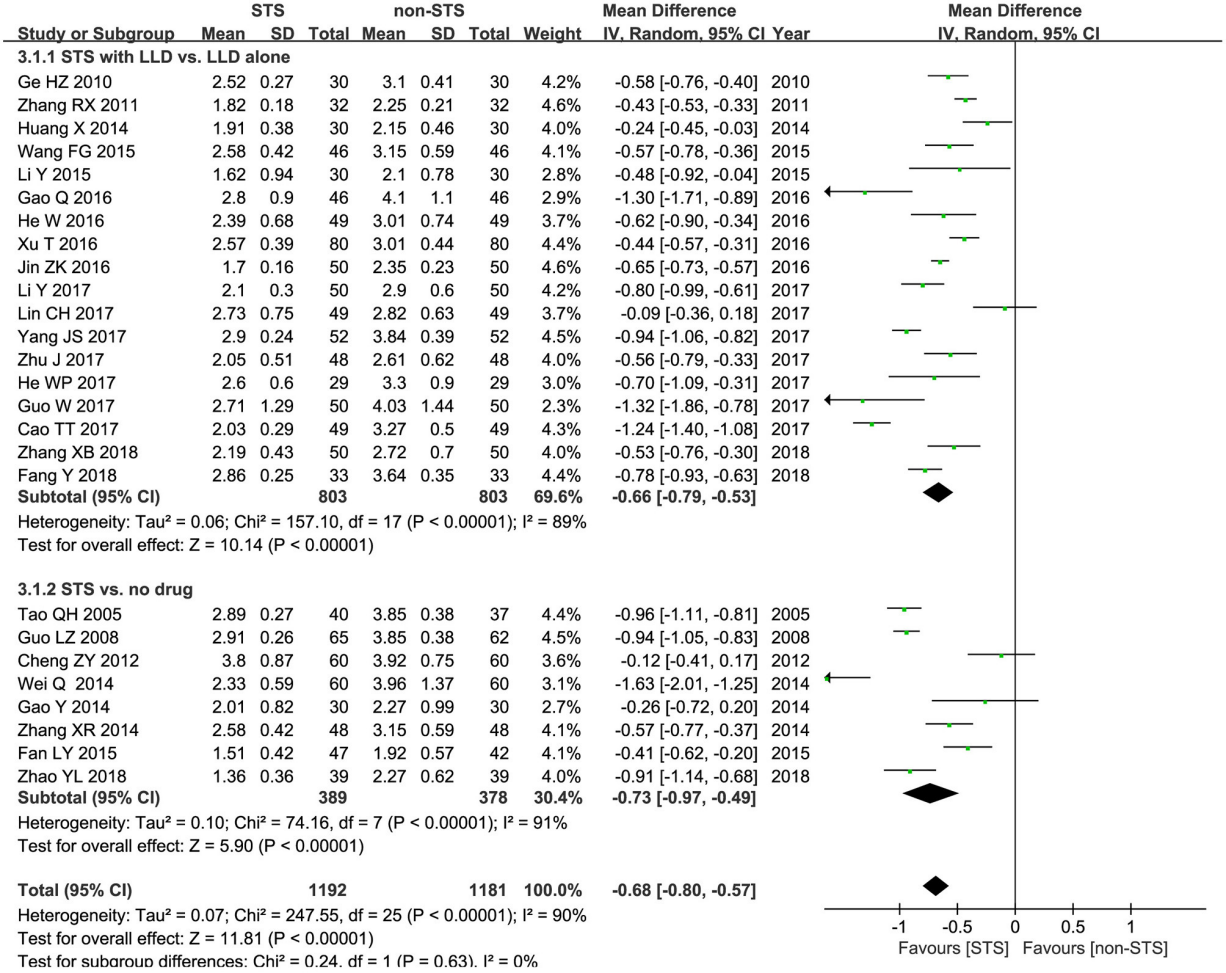
Forest plot of comparison: STS vs. non-STS in LDL-c.

**LDL-c**: Meta-analysis results of 25 studies demonstrated that STS also significantly reduced LDL-c levels in patients with CHD [MD = −0.68 mmol/l, 95% CI (−0.80, −0.57), *P* < 0.00001, *I*^2^ = 96%). Subgroup analysis revealed that STS significantly lowered LDL-c levels when used alone compared to blank control [MD = −0.73 mmol/l, 95% CI (−0.97, −0.49), *P* < 0.00001, *I*^2^ = 91%], or used in combination with LLDs compared to LLDs [MD= −0.66mmol/l, 95% CI (−0.79, −0.53), *P* < 0.00001, *I*^2^ = 89%]. These results are shown in Figure 5.

**Figure 6:**
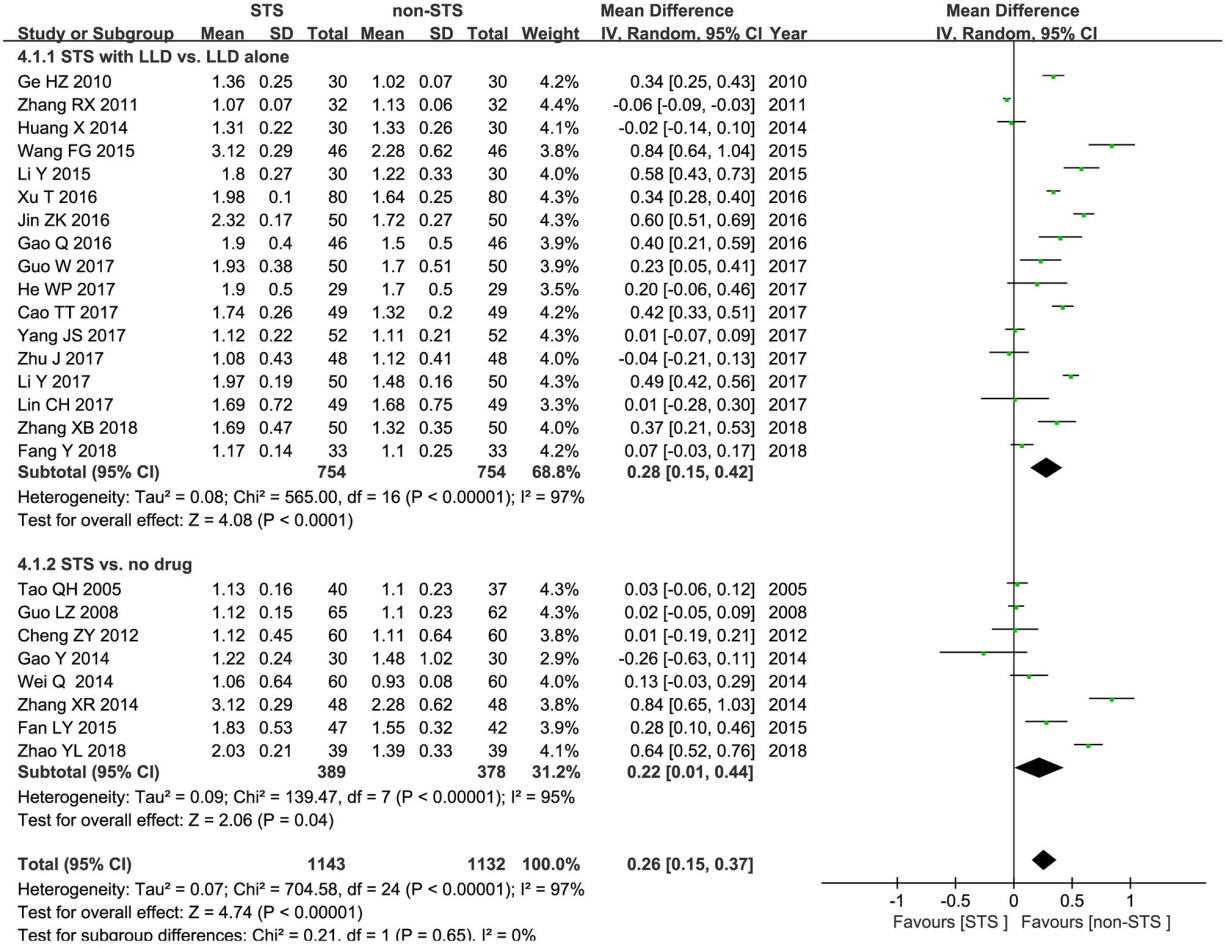
Forest plot of comparison: STS vs. non-STS in HDL-c.

**HDL-c**: Pooled results of 25 RCTs showed that STS significantly increased HDL-c levels [MD = 0.26 mmol/l, 95% CI (0.15, 0.37), *P* < 0.00001, *I*^2^ = 97%]. Subgroup analysis revealed that STS significantly increased HDL-c levels when used alone compared to blank control [MD = 0.22 mmol/l, 95% CI (0.01, 0.44), *P* < 0.00001, *I*^2^ = 95%], or used in combination with LLDs compared to LLDs [MD = 0.28 mmol/l, 95% CI (0.15, 0.42), *P* < 0.00001, *I*^2^ = 97%]. These results are shown in Figure 6.

**Figure 7:**
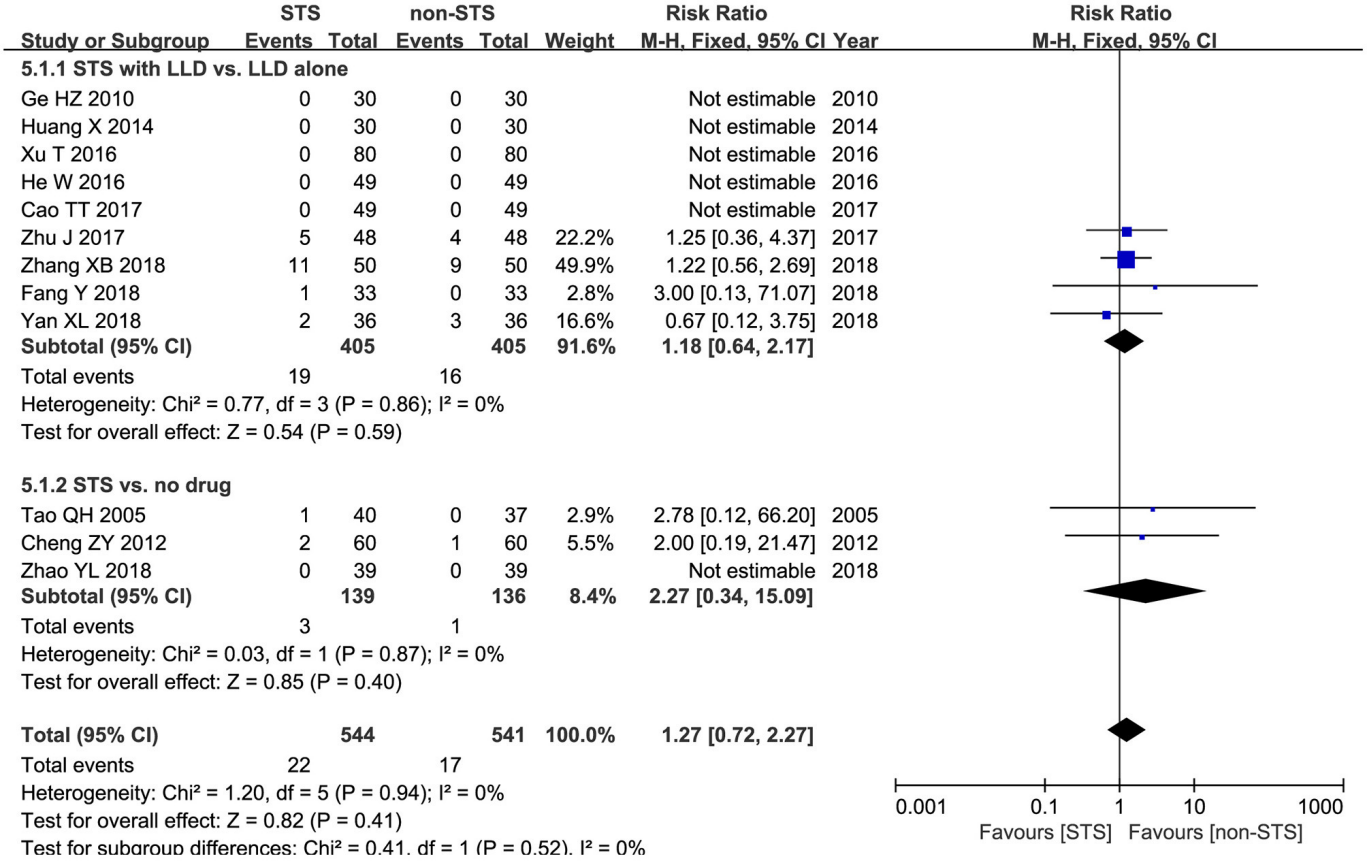
Forest plot of comparison: STS vs. non-STS in AEs.

**Adverse events**: Meta-analysis of 12 studies showed that no statistically significant difference was found between groups in terms of the incidence of adverse events [RR = 1.27, 95% CI (0.72, 2.27), *P* = 0.94, *I*^2^ = 0%]. Subgroup analysis revealed that no statistically significant difference was found between groups in terms of the incidence of adverse events when used alone compared to blank control [RR = −2.27, 95% CI (0.34, 15.09), *P* = 0.94, *I*^2^ = 0%), or used in combination with LLDs compared to LLDs [RR = 1.18, 95% CI (0.64, 2.17), *P* < 0.00001, *I*^2^ = 0%] (Figure 7).

### Meta-Regression

The results above showed that there was considerable heterogeneity (75%-100%) among 27 studies. We conducted a meta-regression analysis which showed that the total dose of STS and the type of statins were important sources of heterogeneity in the included studies (Supplementary Material 2, Supplementary Table S3).

### Sensitivity Analysis

Sensitivity analysis was conducted by excluding the studies from the analysis one by one to understand the impact of this on the results. The results showed that the pooled effects of STS on TC did not change substantially if a single studies were omitted (Supplementary Material 2, Supplementary Figure S1).

### Trial Sequential Analysis

TSA was performed at the level of an overall 5% risk of type I error and a power of 20%. Regarding the results of TSA, shown in the Supplementary Figure S2 (Supplementary Material 2). When the first study was included, the sample size had passed the traditional boundary value and TSA boundary value. After the third study was included, the sample size reached RIS value (RIS value = 220). Although it had reached the statistical threshold, the risk of methodological bias in the original study was high, which may affect the results. It may indicate that the evidence needed to reach a conclusion was sufficient and no further trials were needed.

### Publication Bias

**Figure 8:**
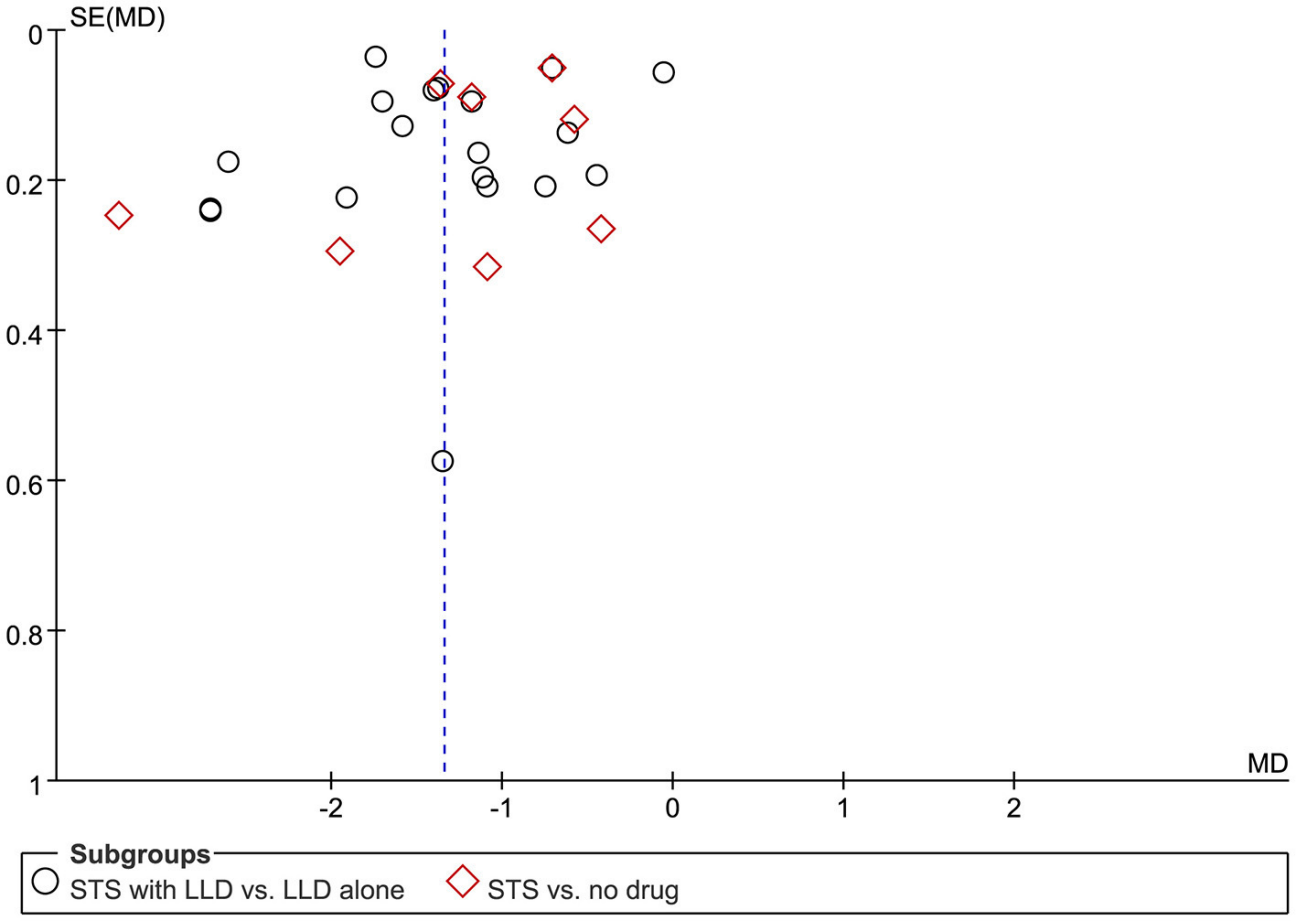
Funnel plot of comparison: STS vs. non-STS in TC.

Figure 8 is a funnel diagram of the impact of STS on TC in patients with CHD, showing asymmetry, indicating possible publication bias. Begge's test and egger's test, respectively obtained *z* = −0.980 (*p* = 0.327) and *t* = −0.702 (*p* = 0.489), indicating that there was no publication bias in statistics. Although the probability of publication bias was statistically small, we still believed that there was a greater possibility of publication bias, because the published studies were all Chinese literature, and the positive results were easier to publish.

## Discussion

In this systematic review, twenty-seven RCTs with a total of 2,445 participants were included to assess the effect of STS on blood lipids in patients with CHD. It showed that STS could significantly reduce the levels of plasma TC, LDL-c and TG, and increase the levels of HDL-c. Through the subgroup analysis we found that the effect of STS was significantly better than control no matter when STS as monotherapy or as adjunctive therapy. At the same time, STS has add-on effect when used in combination with conventional lipid-lowering drugs. In terms of AEs, there was no significant difference between STS group and non-STS group. STS treatment may be safe and effective to reduce the blood lipid level of patients with CHD and has great potential as an assistant drug to reduce blood lipid. However, due to the low overall quality of RCTs at present, the above conclusions need to be verified in larger samples of high-quality research.

Previous studies have mostly focused on the effects of STS on the incidence of major cardiovascular events and inflammatory factors in patients with CHD (64, 65). This systematic review focused for the first time on the effect of STS on blood lipid in patients with CHD and evaluated its safety. At present, there are few studies on the mechanism of STS improving blood lipid level. Researches have shown that STS can effectively reduce LDL-c oxidation in serum and LDL-c level in plasma, which may be related to its peroxy radical scavenging and LDL-c binding activity (66). Tanshinone IIA could increase LDLR levels and LDL uptake by cells through the downregulation of PCSK9 expression in HepG2 cells (67). Tanshinone IIA regulated the SREBP2/PCSK9 pathway, increased the levels of HDL, and decreased lipid deposition in the livers of hyperlipidemic rats (68). On the other hand, tanshinone IIA could lower blood lipid level by downregulating the expression of lipogenic enzymes, including FASN, ACC1, and SCD1, through suppression of SREBP1 expression and LXRα-mediated transcriptional activation, resulting in reducing lipogenesis and lipid accumulation in the HepG2 cells (69). Furthermore, STS can not only affect the changes of AMPK/ACC/CPT1 signal involved in lipid metabolism to inhibit fatty acid beta oxidation during ischemia, but also maintain circulating lipid levels, including TC, TG and free fatty acids (FFA) (70). Moreover, tanshinone IIA is considered to be an inhibitor of apoB secretion and TG secretion in hepatocytes (71) and a natural antagonist of peroxisome proliferator-activated receptor-γ (PPARγ), which is a member of the nuclear receptor superfamily of ligand-activated transcription factors and major transcription factor that regulates the gene expression of adipocyte differentiation, lipogenesis, and glucose metabolism (72). Tanshinone IIA can reduce fat mass and weight, improve glucose tolerance, reduce the ratio of LDL-c to HDL-c without changing food intake (73). In addition, STS can improve the PPAR-alpha down-regulation (74) caused by myocardial infarction and restore insulin signaling pathway (75), thereby improving the hyperlipidemia accumulation of ischemic heart (76).

### Comparison With Previous Studies

Only one systematic review and meta-analysis on the efficacy and safety of Traditional Chinese Medicine injection for blood lipid in patients with unstable angina pectoris (77) was conducted before, in which STS was analyzed, but the results were not described in detail. Our meta-analysis has some strength in comparison with the previous systematic review. First, several newly-published and well-conducted trials were included in our meta-analysis. Second, in the meta-analysis, we performed subgroup analysis from different aspects to make the results more stable.

The results of our meta-analysis are similar to those of the previous review regarding TC and HDL-c when STS was used in combination with LLDs compared to LLDs alone, but they differ with respect to TG and LDL-c. Our results indicated that, compared with LLDs alone, the STS combined with LLDs significantly lowered LDL-c and TG levels. Additionally, we found that STS as monotherapy significantly reduced TG, TC, and LDL-c levels and increased HDL-c levels compared to the controls. Our study carried out a more comprehensive, up-to-date, and PRISMA-compliant systematic review. Meta-analysis provides more reliable clinical evidence.

### Limitation

Although the systematic evaluation shows that STS has a good effect on blood lipid level in patients with CHD, the results are not convincing. Firstly, high-quality, multi-center, large-sample, double-blind RCTs were lacked in the 27 trials included. The 27 trials were not registered in advance, and the relevant test schemes were not published. Secondly, most of the trials did not specify the specific details of random mode, allocation concealment, blind method and so on. This greatly weakens the credibility of the evidence. Finally, the greater heterogeneity and publication bias of the results require us to interpret the final results carefully.

### Implications for Research

The systematic review provides a small amount of evidence for STS in improving the prognosis of patients with CHD. For hospitalized patients with CHD, especially those with statin intolerance, clinicians may consider STS as a complementary alternative therapy. However, the toxicological and pharmacological mechanism of the optimal dose and duration of STS needs further study. Especially for traditional Chinese medicine extracts, we should pay more attention to their adverse reactions to patients. In addition, STS is currently only intravenous preparations, which is obviously not suitable for long-term use of patients. Finding optimum route of administration for STS is another problem we will face in the future. Finally, the design of RCTs and the reporting of clinical study results should be carried out in strict accordance with the requirements of the CONSORT 2010 statement to ensure the scientific quality and rigor of studies (78).

## Conclusion

STS can safely and effectively reduce plasma TC, TG and LDL-c levels in patients with CHD, and improve plasma HDL-c levels. However, these findings require careful recommendation due to the low overall quality of RCTs at present. More multi-center, randomized, double-blind, placebo-controlled trials which are designed follow the CONSORT 2010 guideline are needed.

## Data Availability Statement

The original contributions presented in the study are included in the article/Supplementary Material, further inquiries can be directed to the corresponding authors.

## Author Contributions

QL, HZ, and YZ conceived and drafted this systematic review and registered the protocol at PROSPERO. HZ and QL developed the search strategy and conducted the literature research, study selection, data extraction and risk of rias assessment, and contributed to manuscript drafting. WH, DW, GP, WP, ZW, and XR interpreted the evidence from methodological and clinical perspective. XW oversaw the conduct of the study. All authors have read, critically reviewed, and approved the final manuscript.

## Funding

The research was financially supported by funding from National Natural Science Foundation of China (81774058 and 82074263), National TCM Clinical Research Base operation construction scientific research project of State Administration of Traditional Chinese Medicine (JDZX2015208), and Key Discipline Open Project of Beijing University of Chinese Medicine (2013-ZDXKKF-27).

## Conflict of Interest

The authors declare that the research was conducted in the absence of any commercial or financial relationships that could be construed as a potential conflict of interest.

## Publisher's Note

All claims expressed in this article are solely those of the authors and do not necessarily represent those of their affiliated organizations, or those of the publisher, the editors and the reviewers. Any product that may be evaluated in this article, or claim that may be made by its manufacturer, is not guaranteed or endorsed by the publisher.

## Abbreviations

ACS: Acute Coronary Syndrome
AEs: Adverse Events
AMI: Acute Myocardial Infarction
CCS: Chronic Coronary Syndrome
CHD: Coronary Heart Disease
CI: Confidence Interval
CNKI: China National Knowledge Infrastructure
CONSORT: Consolidated Standards For Reporting Trials
CRP: C-reactive Protein
CVDs: Cardiovascular Diseases
GRADE: grading of recommendations assessment, development and evaluation
HDL-c: High-Density Lipoprotein Cholestero
IL-6: Interleukin-6
LDL-c: Low-density Lipoprotein Cholesterol
LLDs: Lipid-lowering Drugs
MCP-1: Monocyte Chemotactic Protein-1
MD: Mean Difference
MMP-9: Matrix Metalloproteinase-9
NSTE-ACS: Non-ST-elevation Acute Coronary Syndromes
PRISMA: Preferred Reporting Items For Systematic Reviews and Meta-analyses
RCTs: Randomized Controlled Trials
RIS: required information size
RR: Risk Ratio
RT: Routine Therapy
SAMS: Statin-Related Muscle Symptoms
SCAD: Stable Coronary Heart Disease
STS: Sodium Tanshinone IIA Sulfate
TC: Total Cholesterol
TG: Triglycerides
TNF-α: Tumor Necrosis Factor-Alpha
TSA: Trial Sequential Analysis
UAP: Unstable Angina Pectoris.
