# Supplementary material for: Effect of Sodium Tanshinone IIA Sulfonate Injection on Blood Lipid in Patients With Coronary Heart Disease: A Systematic Review and Meta-Analysis of Randomized Clinical Trials
Source: Front Cardiovasc Med. 2021 Nov 24;8:770746. doi: 10.3389/fcvm.2021.770746 (PMC8652084; doi:10.3389/fcvm.2021.770746)
Supplement: Supplementary file 1 [file Presentation_1.zip › Supplementary materials/Supplementary materials 1.docx]

**TableS1 GRADE summary of evidence for effect of STS on Blood Lipid in Patients with CHD**

| **Quality assessment** | | | | | | | **No of patients** | | **Effect** | **Quality** | **Importance** |
| --- | --- | --- | --- | --- | --- | --- | --- | --- | --- | --- | --- |
|  |  |  |  |  |  |  |  |  |  |  |  |
| **(outcomes)No of studies** | **Design** | **Risk of bias** | **Inconsistency** | **Indirectness** | **Imprecision** | **Other considerations** | **STS** | **non-STS** | **Absolute** |  |  |
| (TC) 27 | randomised trials | serious | very serious | no serious indirectness | no serious imprecision | none | 1228 | 1217 | MD 1.34 lower (1.59 to 1.09 lower) | ⊕OOO VERY LOW | IMPORTANT |
| (TG) 26 | randomised trials | serious | very serious | no serious indirectness | no serious imprecision | none | 1198 | 1187 | MD 0.49 lower (0.62 to 0.35 lower) | ⊕OOO VERY LOW | IMPORTANT |
| (LDL-c) 26 | randomised trials | serious | very serious | no serious indirectness | no serious imprecision | none | 1192 | 1181 | MD 0.68 lower (0.8 to 0.57 lower) | ⊕OOO VERY LOW | IMPORTANT |
| (HDL-c) 25 | randomised trials | serious | very serious | no serious indirectness | no serious imprecision | none | 1143 | 1132 | MD 0.26 higher (0.15 to 0.37 higher) | ⊕OOO VERY LOW | IMPORTANT |

**TableS2 GRADE summary of evidence for safety of STS on Patients with CHD**

| **Quality assessment** | | | | | | | **No of patients** | | **Effect** | | **Quality** | **Importance** |
| --- | --- | --- | --- | --- | --- | --- | --- | --- | --- | --- | --- | --- |
|  |  |  |  |  |  |  |  |  |  |  |  |  |
| **(outcomes)**  **No of studies** | **Design** | **Risk of bias** | **Inconsistency** | **Indirectness** | **Imprecision** | **Other considerations** | **STS** | **non-STS** | **Relative (95% CI)** | **Absolute** |  |  |
| (AEs) 12 | randomised trials | serious | no serious inconsistency | serious | no serious imprecision | none | 22/544  (4%) | 17/541  (3.1%) | RR 1.27 (0.72 to 2.27) | 8 more per 1000 (from 9 fewer to 40 more) | ⊕⊕OO LOW | IMPORTANT |
