# Supplementary material for: Effect of Sodium Tanshinone IIA Sulfonate Injection on Blood Lipid in Patients With Coronary Heart Disease: A Systematic Review and Meta-Analysis of Randomized Clinical Trials
Source: Front Cardiovasc Med. 2021 Nov 24;8:770746. doi: 10.3389/fcvm.2021.770746 (PMC8652084; doi:10.3389/fcvm.2021.770746)
Supplement: Supplementary file 1 [file Presentation_1.zip › Supplementary materials/Supplementary materials 2.docx]

**Table S3 Meta-regression of the association between TC in patients with CHD, and the total dose of STS and the types of statins**

|  | β | 2.5% CI | 97.5%CI | *p* vlue |
| --- | --- | --- | --- | --- |
| Total dose of STS^*^ |  |  |  |  |
| >750mg | -0.787 | -1.25 | -0.32 | 0.001 |
| Types of statins^#^ |  |  |  |  |
| nonstatin | 0.392 | -0.19 | 0.97 | 0.185 |
| Simvastatin | 0.892 | 0.14 | 1.64 | 0.020 |
| statin | 0.594 | 0.05 | 1.14 | 0.034 |

* Total dose of STS is mean that product of daily dose of STS and treatment duration.

# Atorvastatin as a dummy variable.


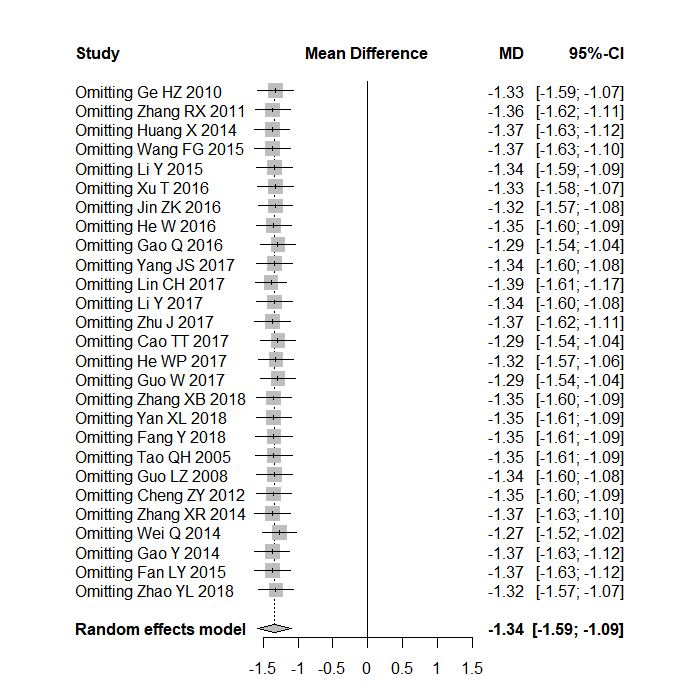


**Figure S1 Sensitivity analysis of TC in patients with CHD treated by STS**

**
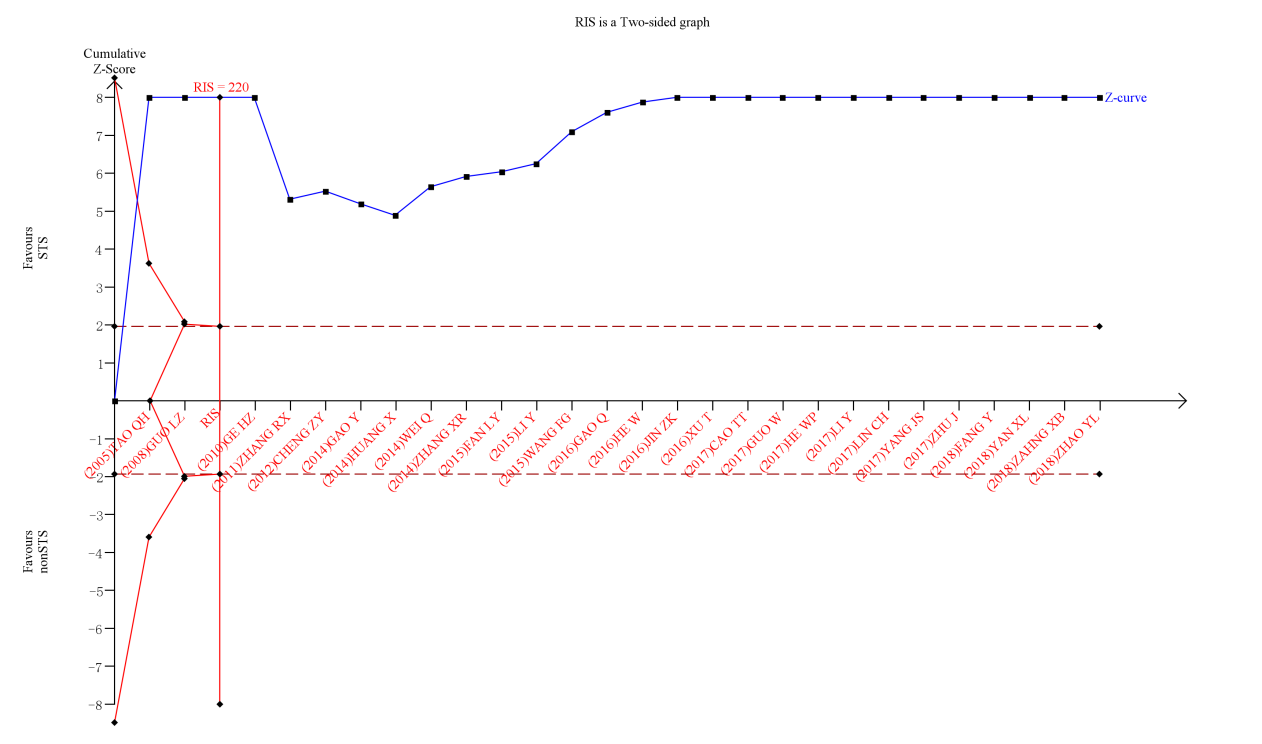
**

**Figure S2 Trial Sequential Analysis (TSA) on pooled result of the effects of STS on TC of this meta-analysis**
